# Inter-rater and test-retest reliability of movement control tests for the neck, shoulder, thoracic, lumbar, and hip regions in military personnel

**DOI:** 10.1371/journal.pone.0204552

**Published:** 2018-09-25

**Authors:** Matthias Tegern, Ulrika Aasa, Björn O. Äng, Karin Harms-Ringdahl, Helena Larsson

**Affiliations:** 1 Department of Community Medicine and Rehabilitation, Physiotherapy, Umeå University, Umeå, Sweden; 2 Department of Neurobiology, Care Sciences and Society, Division of Physiotherapy, Karolinska Institutet, Huddinge, Sweden; 3 School of Education, Health and Social Studies, Dalarna University, Falun, Sweden; 4 Allied Health Professionals Function, Functional area Occupational therapy and Physiotherapy, Karolinska University Hospital, Stockholm, Sweden; 5 Swedish Armed Forces, HQ, Stockholm, Sweden; Szegedi Tudomanyegyetem, HUNGARY

## Abstract

Strategies are needed to mitigate the high rates and related risks of musculoskeletal complaints and injuries (MSCI) in the military aviator community. Previous work on Swedish Armed Forces (SwAF) soldiers have shown that proper screening methods have been successful in reducing early discharge from military training. Research has pointed at the importance of optimal spinal movement control in military aviators. The aim of this work was to investigate the inter-rater and test-retest reliability of a battery of clinical tests for evaluating movement control in the neck, shoulders, thoracic, lumbar, and hip regions in a population of SwAF military personnel. Inter-rater and test-retest reliability of 15 movement control tests were assessed by crude and prevalence-adjusted kappa coefficient. The study included 37 (inter-rater) and 45 (test-retest) SwAF personnel and was performed with two physiotherapists simultaneously observing and rating the movements on the first occasion and repeated with one physiotherapist on the second occasion. For inter-rater reliability, the kappa coefficient ranged from .19 to .95. Seven tests showed substantial to almost perfect agreement (kappa > .60). With the adjusted kappa, three more tests reached the level of substantial agreement. The corresponding values for test-retest reliability ranged from .26 to .65. Substantial agreement was attained for two tests, three with adjusted kappa. The following tests can reliably be used when screening for biomechanically less advantageous movement patters in military aviators: Shoulder flexion, and rotation, Neck flexion in sitting and supine, Neck extension and rotation in sitting, Pelvic tilt, Forward lean and Single and Double knee extension tests. Grading criteria for tests in supine and quadruped positions need to be further elaborated.

## Introduction

Musculoskeletal pain is the main cause of disability in most countries [1]. In the general population, the low back [2] and the neck [3] are the most commonly affected regions. This is also true for military aviators, including fighter [4–6] and helicopter [7, 8] pilots as well as helicopter cabin crew [9]. In active military ground units, the highest prevalence of musculoskeletal pain is in the low back and the lower extremities [10–12]. Musculoskeletal injuries cause a higher rate of lost duty days than illness. When soldiers have regular episodes of musculoskeletal complaints, this might affect a unit’s readiness [12]. Prevention of musculoskeletal injuries is thus important.

As a preventive method, screening for early signs of musculoskeletal complaints can be useful. In the Swedish Armed Forces (SwAF), the Musculoskeletal Screening Protocol (MSP) targeting early signs of Musculoskeletal Complaints and Injuries (MSCI) has been successful. The MSP includes a questionnaire, tests for muscular capacity and loading tests for the knee joint. By using this protocol, soldiers that need further examination can be identified and go through an assessment and receive individualized treatment for their MSCI. Together, the MSP and individualized interventions have significantly reduced the numbers of early discharged soldiers due to MSCI [10, 13, 14]. However, there is currently no specific screening tool for neck or back pain, which is especially important for SwAF aviators. Since research including military aviators has pointed out the importance of also including screening of movement control [5, 15], it has been suggested that the screening protocol should not only focus on muscular strength and endurance but also on the presence of non-optimal movement control leading to biomechanically less advantageous movement patterns [15–19]. Altered neuromuscular movement control has been mentioned to be a key component in the recurrence of neck pain episodes [20]. Underlying these assumptions is the kinesiopathological theory hypothesis that the use of non-ideal alignments or biomechanically less advantageous movement patterns over time will cause tissue irritation and eventually cause mechanical neck and back pain. This implies that it is crucial to identify early signs of non-ideal postures and movement patterns [16, 19, 21].

Reliable screening tests are needed to add to existing MSP questionnaires to identify early signs of MSCI in SwAF aviators. The inter-rater reliability has been found to be at least substantial (i.e. Kappa >0.6) for the majority of clinical movement control tests previously studied using videos [22–25] or specific items from physical examinations [26] in patients with neck or low back pain. Video analysis has further been used to determine intra-rater reliability of movement control tests, where the same movements have been rated at two defined occasions with generally high reliability, i.e., thus with no subject variability from test to retest [22, 25]. Monnier et al. investigated both inter-rater and test-retest reliability of six movement control tests with dichotomous outcome (i.e. pass or fail) for the low back using two raters examining SwAF marines on two different occasions using live observations [27]. Their findings suggested that inter-rater reliability was moderate to almost perfect, while the reproducibility on test-retest examination was fair to moderate, indicating variability between occasions among the subjects.

Reliability studies are ideally performed on the target population because reliability for clinical tests might be specific to the group under study, including differences in disease prevalence. Due to ongoing studies conducted on SwAF aviators, the inclusion of this population was prohibited. However, statistical solutions that control for prevalence bias can be used to handle these situations [28]. To our knowledge, no previous study has investigated inter-rater and test-retest reliability of movement control tests targeting both the neck, shoulder, thoracic, lumbar, and hip regions, in military personnel using live observations. Therefore, the aim of this work was to investigate the inter-rater and test-retest reliability of a battery of clinical tests for evaluating movement control in the neck, shoulders, thoracic, lumbar, and hip regions in a population of military personnel.

## Materials and methods

### Study design

An inter-rater and test-retest [29] reliability study of movement control tests was performed. A power analysis revealed that the sample size needed was 35 participants with an agreement of 90%, a confidence interval (CI) of 20%, and a chance agreement of 50% [30]. Participants provided written consent prior to the first test occasion. The individual displayed in this manuscript has given written informed consent (as outlined in PLOS consent form) to publish the pictures. The Regional Medical Research Ethics Committee in Stockholm approved the study (DNR:2012/1690-32).

### Participants

All participants were employed within the SwAF at three different workplaces, and their characteristics are presented in Table 1. Their specific occupations were as soldiers or as non-soldiers (i.e. officers or civilians) working with other tasks such as administration or education. An introductory letter was posted at the different workplaces with the possibility to voluntarily participate in the study. In the inter-rater reliability part, a total of 37 participants were tested, and in the test-retest reliability part, a total of 48 participants were tested on the first occasion. Three participants were lost to the second test occasion due to sick leave, therefore 45 participants were included in this analysis. In total, 25 persons (all from the same workplace) were included in both the inter-rater and test-retest analysis.

**Table 1:** Participant characteristics for the inter-rater (n = 37) and test-retest (n = 45) studies.

	Inter-rater	Test-retest
	Mean (SD)	Mean (SD)
Age (years)	35.8 (10.3)	35.0 (10.4)
Body Mass Index (BMI) (kg/m^2^)	24.9 (2.4)	24.5 (2.4)
Height (m)	1.78 (0.1)	1.78 (0.1)
Weight (kg)	79.4 (13.4)	78.0 (12.7)
Sex (Male/Female %)	62/38	71/29
Occupation (Soldier/Non-soldier %)	49/51	49/51

SD, standard deviation

### Procedure

At the day of the data collection, we followed the standardized procedure from the Musculoskeletal Screening Protocol (MSP), therefore all participants answered a short form of the MSP questionnaire. Two experienced physiotherapists (PT1 and PT2) performed the testing. Both had been working as PTs for more than 9 years using movement control tests in their clinical work and treating patients with musculoskeletal complaints daily in the SwAF or in primary health care. They were well familiarized with the standardizations and descriptions of each of the tests included in the study.

Immediately prior to the study, the PTs met for 2 days to review the test protocol with the description of movements and to practice the test protocol on several volunteers (not included in the study), and minor amendments to the protocol were made. In the inter-rater part, PT1 and PT2 rated the performance simultaneously but on separate sheets using live observations without communicating with each other. PT1 instructed all participants and performed the re-test on average 5.3 days later (min-max: 2–7 days) in the test-retest part.

### Test protocol

The test protocol included 15 movement control tests, of which six of the tests were performed for the left and right extremities or neck rotation to the left or right for a total of 21 movements (for an overview see Table 2 and a detailed description including grading criteria, see S1 Table). The tests are commonly used in clinical practice to identify non-ideal movement patterns [16, 31] or the inability to dissociate movement in one joint from an adjacent joint [18]. Prior to each test, a short video of the test was shown to the participants together with verbal instructions given by PT1. The participants repeated the movement three times to ensure familiarization with the movement to be tested, thereafter they performed the movement and the PTs rated them as either “optimal” or “non-optimal” movement patterns (i.e. “pass” or “fail” of the test). In the case of a non-optimal rating, the PTs noted the reason according to the pre-defined grading criteria (S1 Table). The reason was noted in the protocol but not included in the reliability analysis. No feedback regarding test outcome was given during or after the test. The order of the tests was maintained throughout the study, beginning with tests involving standing followed by sitting, supine, and quadruped test positions.

**Table 2:** Summary of tests included in the protocol, the purpose of each test, and the position for the tests.

Test	Purpose	Side	Position
**Shoulder region**			
Shoulder flexion test	To assess the ability to move the arm into flexion to about 180° with ≈60 °upward rotation and no excessive winging, excessive elevation/abduction/forward tilt/downward rotation of the scapula or medial rotation of the humerus.	L/R	Standing
Shoulder extension test	To assess the ability to extend the arm to about 15° while retaining a neutral position of the scapula.	L/R	Standing
Shoulder lateral rotation test	To assess the ability to laterally rotate the shoulder to about 45° while retaining a neutral position of the scapula.	L/R	Standing
**Neck region**			
Neck flexion in sitting test	To assess the ability to flex the neck to 45°–50° with contribution of both lower (≈35°) and upper cervical spine without cervical anterior translation/diminished anterior sagittal plane rotation.	-	Sitting
Neck extension in sitting test	To assess the ability to extend the neck to ≈85° with contribution of both lower (≈70°) and upper cervical spine without mid-cervical anterior translation.	-	Sitting
Neck rotation test	To assess the ability to rotate the cervical spine to ≈70°–80° without concurrent neck or shoulder movements.	L/R	Sitting
Neck flexion in supine test	To assess the ability to smoothly flex the neck using all cervical segments without excessive anterior translation.	-	Supine
Neck extension in quadruped test	To assess the ability to smoothly extend the cervical spine using all cervical segments without excessive posterior translation.	-	Quadruped
**Thoracic, lumbar and hip region**			
Chest lift test	To assess the ability to extend the thoracic spine (lifting the chest) without anterior pelvic tilt and lumbar extension.	-	Sitting
Pelvic tilt test	To assess the ability to tilt the pelvis posteriorly without thoracic flexion.	-	Sitting
Forward lean test	To assess the ability to flex the hip and to lean forward to about 30° without lumbar flexion.	-	Sitting
Single knee extension test	To assess the ability to extend the knee to about 10°–15° from full extension without lumbar flexion or rotation.	L/R	Sitting
Double knee extension test	To assess the ability to extend both knees to about 10°–15° from full extension without lumbar flexion.	-	Sitting
Leg lift test	To assess the ability to flex the hip joint to about 120° without lumbar flexion or posterior pelvic tilt.	L/R	Supine
Rocking forward test	To assess the ability to extend the hips to about 0° in quadruped position without lumbar extension.	-	Quadruped

L/R = Test performed on both the left and right side.

### Questionnaire

The short form of the MSP questionnaire includes questions about sex, age, height, weight and presence of Musculoskeletal Complaints and Injuries (MSCI): “*Have you had any musculoskeletal complaints or injuries during the last year*?” and “*Do you still have these at present*?”. The participants’ current pain intensity was rated on a 0–100 mm visual analogue scale for 10 anatomical locations. A rating of 0 represented “no pain at all” and 100 the “worst imaginable pain” [10]. The questionnaire was answered after both test 1 and test 2 (in the test-retest part) and was administered by an independent research assistant. The assistant instructed the participants not to reveal any information if they had any MSCI during the testing.

### Data analysis

The percentage agreement and the kappa coefficient with 95% CI was used to analyse the inter-rater reliability (between PT1 and PT2) and test-retest reliability (PT1’s rating of test 1 and test 2) of the movement control tests. The kappa coefficient is a used to quantify agreement beyond chance [32] but is susceptible to prevalence and bias, and thus the prevalence-adjusted bias-adjusted kappa coefficients (PABAK) aided the interpretation of reliability [28]. The strength of agreement is commonly interpreted as follows: <0 poor agreement, .01–.20 slight agreement, .21–.40 fair agreement, .41–.60 moderate agreement, .61–.80 substantial agreement, and .81–.99 almost perfect agreement [32]. Data analyses were performed using IBM SPSS version 22 (IBM Inc.).

The McNemar test was used to evaluate any possible learning effect from test 1 to test 2 by comparing the number of passed tests on test 1 to the number of passed tests on test 2. A significance level of .05 was used.

## Results

**Fig 1:**
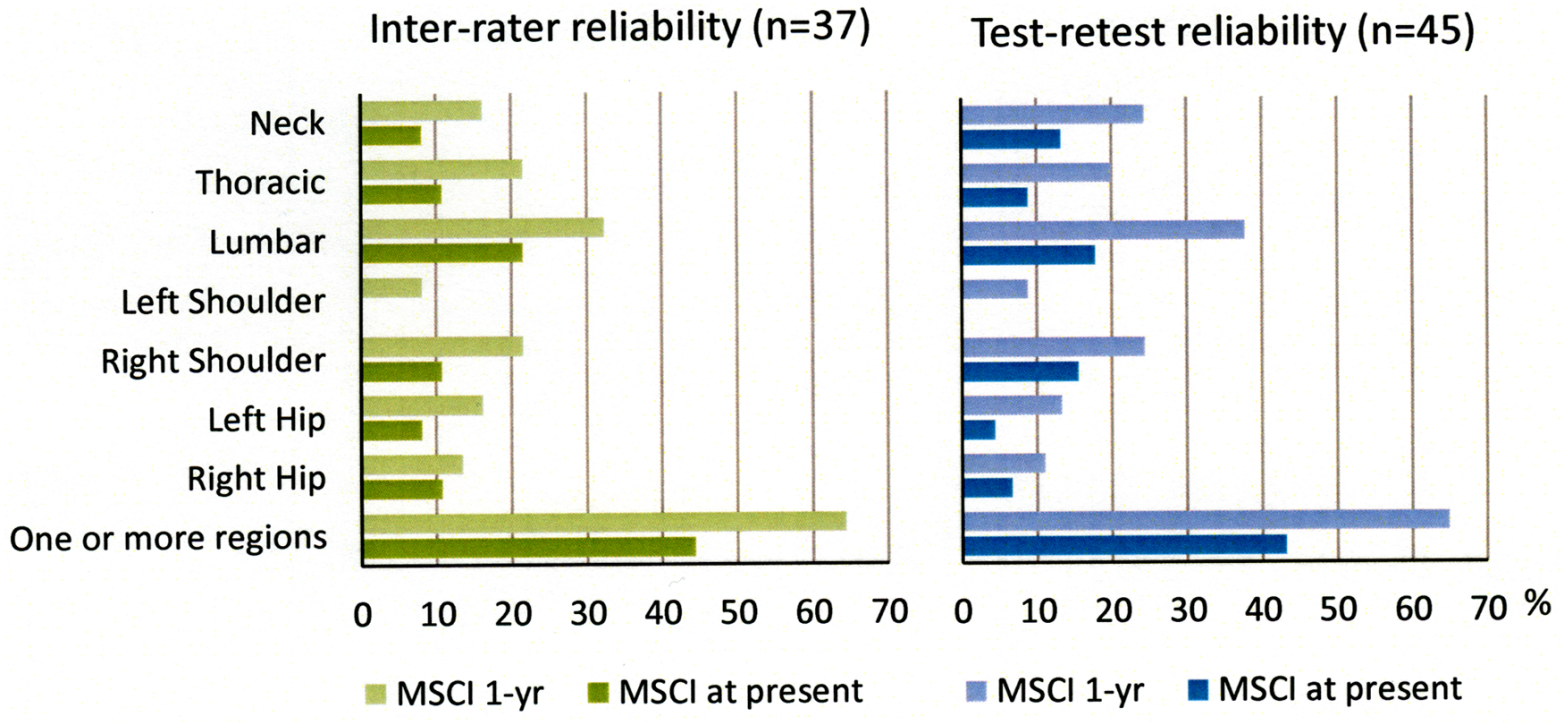
Percentage-distribution of prevalence of musculoskeletal complaints or injuries (MSCI). MSCI among the participants in the inter-rater and test-retest study. Percentages are presented for MSCI during the last year (1-yr) or MSCI at present for each of the investigated regions and overall in one or more regions.

Fig 1 shows that many of the military personnel reported MSCI; the highest one-year prevalence was 32.4% and 37.8% for MSCI in the lumbar spine for both the inter-rater and the test-retest study groups, respectively (the corresponding MSCI at present was 21.6% and 17.8%, respectively). For any (one or more region) MSCI the one year prevalence was 64.4% and 64.9%, and for MSCI at present the prevalence was 44.4% and 43.2%, respectively. The secondary analyses showed that there were no significant differences between the number of passed tests between participants with ongoing or previous MSCI and those without MSCI. (7) Further, there was no significant difference in the number of tests with a changed outcome (e.g. optimal on test 1 but non-optimal on test 2) from test 1 to test 2 between participants with and without MSCI, respectively. The within-group McNemar test showed no significant difference on any movement control test from test 1 to test 2, indicating no learning effect among the participants.

The results of inter-rater and test-retest reliability of the 15 movement control tests are shown in Tables 3 and 4, respectively. The percentage agreement ranged from 60% to 97% and the kappa coefficient ranged from .19 to .95 for inter-rater reliability. The tests with kappa >.60, indicating substantial to almost perfect agreement, were Shoulder flexion, Shoulder lateral rotation (right), Neck rotation, Neck flexion in supine, Forward lean, Single knee extension, and Double knee extension. The corresponding values for test-retest reliability ranged from 64% to 84% and kappa from .26 to .65, and Pelvic tilt and Single knee extension (right) attained substantial agreement. By calculating PABAK, Neck flexion in sitting, Neck extension in sitting, and Pelvic tilt attained substantial agreement (from kappa = .44–.57 to .62–.78) for inter-rater reliability. Regarding test-retest reliability, Neck rotation (right) attained substantial agreement (from kappa = .60 to.64).

**Table 3:** Inter-rater reliability of movement control tests and the number of passed and failed tests (n = 37).

Movement control tests		%-	κ	95%	SE	Strength	PABAK	95%	SE	Strength	Passed/Failed (n)	
		agreement		CI				CI			PT1	PT2
**Shoulder region**												
Shoulder flexion	left^1^	89.2	.78	.58-.98	.10	Substantial	.78	.58-.98	.10	Substantial	20/17	22/15
	right^1^	97.3	.95	.84–1.05	.05	Almost Perfect	.95	.84–1.05	.05	Almost Perfect	21/16	20/17
Shoulder extension	left	70.3	.40	.10-.69	.15	Fair	.41	.11-.70	.15	Moderate	16/21	17/20
	right	75.7	.44	.13-.74	.16	Moderate	.51	.24-.79	.14	Moderate	13/24	10/27
Shoulder lat. rotation	left	73.0	.47	.19-.74	.14	Moderate	.46	.17-.60	.15	Moderate	20/17	20/17
	right^1^	83.8	.68	.45-.91	.12	Substantial	.68	.44-.91	.12	Substantial	20/17	16/21
**Neck region**												
Neck flexion in sitting^2^		89.2	.44	.02-.90	.23	Moderate	.78	.58-.98	.10	Substantial	4/33	4/33
Neck extension in sitting ^2^		81.1	.57	.30-.84	.14	Moderate	.62	.37-.87	.13	Substantial	9/28	14/23
Neck rotation	left^1^	89.2	.71	.44-.97	.14	Substantial	.78	.58-.98	.10	Substantial	10/27	8/29
	right^1^	83.8	.64	.39-.90	.13	Substantial	.68	.44-.91	.12	Substantial	13/24	13/24
Neck flexion in supine ^1^		91.9	.84	.66–1.01	.09	Almost Perfect	.84	.66–1.01	.09	Almost Perfect	16/21	17/20
Neck extension in quadruped		59.5	.19	-.13-.50	.16	Slight	.19	−.13-.51	.16	Slight	17/20	18/19
**Thoracic, lumbar, and hip region**												
Chest lift		78.4	.48	.18-.79	.16	Moderate	.57	.30-.83	.14	Moderate	12/25	10/27
Pelvic tilt^2^		89.2	.44	-.02-.90	.23	Moderate	.78	.58-.98	.10	Substantial	4/33	4/33
Forward lean^1^		83.8	.66	.42-.90	.12	Substantial	.68	.44-.91	.12	Substantial	25/12	21/16
Single knee extension	left^1^	89.2	.78	.59-.98	.10	Substantial	.78	.58-.98	.10	Substantial	17/20	19/18
	right^1^	83.8	.65	.42-.89	.12	Substantial	.68	.44-.91	.12	Substantial	10/27	16/21
Double knee extension^1^		86.5	.69	.44-.94	.13	Substantial	.73	.51-.95	.11	Substantial	13/24	10/27
Leg lift	left	78.4	.57	.32-.83	.13	Moderate	.57	.30-.83	.14	Moderate	21/16	17/20
	right	75.7	.49	.21-.76	.14	Moderate	.51	.24-.79	.14	Moderate	16/21	11/26
Rocking forward		62.2	.23	-.01-.47	.12	Fair	.24	−.07-.56	.16	Fair	18/19	5/32

κ, kappa coefficient: 95% CI, 95% confidence interval: SE, standard error of kappa: Strength, agreement according to Landis and Koch (1977): PABAK, Prevalence-Adjusted Bias-Adjusted Kappa: PT, physiotherapist.

^1^Tests with kappa coefficient ≥ .60.

^2^Tests with kappa coefficient ≤ .60 and corresponding PABAK > .60.

**Table 4:** Test-retest reliability of movement control tests and the number of passed and failed tests (n = 45).

Movement control tests		%-	κ	95%	SE	Strength	PABAK	95%	SE	Strength	Passed/Failed (n)	
		agreement		CI				CI			PT1 Test 1	PT1 Test 2
**Shoulder region**												
Shoulder flexion	left	66.7	.34	.06-.61	.14	Fair	.33	.06-.61	.14	Fair	25/20	22/23
	right	77.8	.55	.31-.79	.12	Moderate	.56	.31-.80	.12	Moderate	27/18	23/22
Shoulder extension	left	75.6	.51	.26-.76	.13	Moderate	.56	.31-.80	.12	Moderate	23/22	24/21
	right	64.4	.26	-.02-.55	.15	Fair	.29	.02-.56	.14	Moderate	19/26	17/18
Shoulder lat. rotation	left	77.8	.54	.30-.79	.13	Moderate	.56	.31-.60	.12	Moderate	25/20	26/19
	right	80.0	.58	.33-.82	.13	Moderate	.60	.37-.83	.12	Moderate	27/18	28/17
**Neck region**												
Neck flexion in sitting		68.9	.32	.06-.59	.14	Fair	.38	.11-.65	.14	Fair	11/34	19/26
Neck extension in sitting		75.6	.36	.06-.66	.16	Fair	.56	.31-.80	.12	Moderate	13/32	10/35
Neck rotation	left	66.7	.32	.04-.59	.14	Fair	.33	.06-.61	.14	Fair	20/25	17/28
	right^2^	82.2	.60	.36-.85	.12	Moderate	.64	.42-.87	.11	Substantial	17/28	13/32
Neck flexion in supine		73.3	.41	.13-.68	.14	Moderate	.47	.21-.72	.13	Moderate	17/28	13/32
Neck extension in quadruped		66.7	.30	.01-.58	.15	Fair	.33	.06-.61	.14	Fair	18/27	17/28
**Thoracic, lumbar, and hip region**												
Chest lift		80.0	.57	.32-.82	.13	Moderate	.60	.37-.83	.12	Moderate	17/28	16/29
Pelvic tilt^1^		84.4	.65	.41-.88	.12	Substantial	.69	.48-.90	.11	Substantial	13/32	16/29
Forward lean		71.1	.39	.12-.66	.14	Fair	.42	.15-.69	.14	Moderate	31/14	26/19
Single knee extension	left	73.3	.38	.09-.67	.15	Fair	.47	.21-.72	.13	Moderate	15/30	13/32
	right^1^	84.4	.63	.39-.88	.13	Substantial	.69	.48-.90	.11	Substantial	15/30	12/33
Double knee extension		77.8	.53	.27-.78	.13	Moderate	.56	.31-.80	.12	Moderate	16/29	18/27
Leg lift	left	77.8	.55	.31-.80	.12	Moderate	.56	.31-.80	.12	Moderate	25/20	23/22
	right	66.7	.27	-.01-.55	.14	Fair	.33	.06-.61	.14	Fair	18/27	13/32
Rocking forward		71.1	.42	.16-.69	.13	Moderate	.42	.15-.69	.14	Moderate	20/25	23/22

κ, kappa coefficient: 95% CI, 95% confidence interval: SE, standard error of kappa: Strength, agreement according to Landis and Koch (1977): PABAK, Prevalence-Adjusted Bias-Adjusted Kappa: PT, physiotherapist: Test 1, first test: Test 2, second test.

^1^Tests with kappa coefficient ≥ .60.

^2^Tests with kappa coefficient ≤ .60 and corresponding PABAK > .60

The within-group McNemar test showed no significant difference on any movement control test from test 1 to test 2, indicating no learning effect among the participants.

## Discussion

This is the first study to investigate inter-rater and test-retest reliability of movement control tests for several body regions, in a military population. The inter-rater reliability for seven of the 15 tests reached a level representing substantial to almost perfect agreement (kappa = .64–.95). With PABAK, three more tests reached the level of substantial agreement (kappa > .60). The test-retest results showed generally lower kappa coefficients than the inter-rater values, and two tests (three when PABAK was calculated) showed substantial agreement (test-retest kappa = .63–.65). These two tests also had acceptable results on inter-rater reliability. Namely, the Single knee extension (right side) had an inter-rater kappa of .65 and the Pelvic tilt test had a moderate inter-rater kappa of .44, but a substantial PABAK of .78. The third test was Neck rotation (right side) had an inter-rater kappa of .64. The difference between PABAK and unadjusted kappa coefficients was due to an unequal prevalence of passed/failed results on these tests.

The kappa coefficients for inter-rater reliability ranged from .19 to .95 and are in line with previous results of studies including movement control tests for the low back [22, 27] and neck regions [25], but they are somewhat lower than in two studies including tests of the neck and shoulder region [23, 24]. Notably, all above-mentioned studies but one [27] used video recordings, where the same movement can be observed several times. The present Shoulder flexion test (left side, kappa = .95) and the Neck flexion in supine test (kappa = .84) showed almost perfect agreement, and the latter test was comparable to previous findings [24]. The Rocking forward test and the Neck extension in quadruped test were the tests with the lowest reliability (kappa < .23). The reason why some tests showed low inter-rater reliability might be due to the grading criteria. For these two tests, it might have been difficult to evaluate if the levator scapulae muscle was overactive during neck extension or if there is an uncontrolled lumbar extension during rocking forward in quadruped positions. Another reason might be that the PTs were less familiar with rating tests in other positions than sitting and standing. The fact that tests performed in the quadruped position earlier have shown fair to moderate agreement [22, 25] may support this: in Luomajoki et al. [22], the Rocking forward in quadruped position test showed moderate agreement (kappa = .45) when a pair of less experienced raters judged the movement. Further, Segarra et al. [25] found that two other tests in this position had fair to moderate agreement (kappa = .36 and .52, respectively). As in line with the discussions by Luomajoki et al. (23), the two PTs in our study may have benefited from a longer period of practice focusing on tests in unfamiliar positions. This might have increased the percentages of agreement and kappa coefficients [22].

Regarding the test-retest kappa coefficients, the attained values (kappa = .26–.65) are in line with another study using the same design as we used with live observations on both occasions. Monnier et al. studied movement control of the low back among Swedish Marines with kappa coefficients ranging from .22 to 58. It is known that test-retest values might vary due to the instrument itself, the tester, the person being tested, or the circumstances under which the measurements are taken [29]. The reason for using live observations was to enhance ecological validity. However, when using live observations, there is always a risk for a learning effect or that other modifying factors would affect the movement pattern. Therefore we chose a time interval of a minimum of two and a maximum of seven days to minimize these risks [28]. There was no increase in number of passed tests from test 1 to test 2, indicating no learning effects from such repeated testing with these tests. This was also shown for 5 out of 6 tests in the study by Monnier et al. [27]. However, the participants might still have moved differently on test 1 and test 2. Regarding modifying factors, we were not able to control the participants´ activities between test 1 and test 2 because we included active SwAF personnel. It is possible that their training activities might have influenced active range of motion in the examined regions and thereby have induced variability in the test-retest results. However, the fact that earlier studies using video-recordings have shown satisfactory reliability [22, 25] indicates that experienced clinicians can assess movement control in the neck as well as the back fairly reliably if the movement is performed similarly as is the case when the same video-recordings are being analysed on two separate occasions. Though, in our study, it cannot be excluded that PT1 judged the movements differently on test 1 and test 2. There might have been subtle movement deviations that were hard to judge based on the grading criteria to rate pass or fail (i.e. optimally or non-optimally performed movement) especially since palpation was not allowed. In the clinic, palpation is often part of the evaluation of the muscle recruitment patterns during the movements [21, 26, 33] and is also described in the original tests [16, 18, 31]. These subtle deviations may be explained by the dynamical systems theory, describing that there is a large variability in how movements can be performed [34]. Motor variability is the natural variation of the way a movement can be performed because we adapt to changes in the external environment and to variations in internal physiology in order to ensure a motor solution to these changes [35]. Further, it has been found that there is significant motor variability among healthy persons performing a repetitive task [36]. Movement variation has also been shown to be present in individuals with acute pain, whereas in individuals with longer pain duration the amount of variation is reduced [35].

To easier interpret the kappa coefficients, and to enhance the generalizability of our results, prevalence and bias adjusted kappa coefficients (PABAK) were calculated. We consider the results to mainly be interpreted for persons without or with at most mild to moderate MSCI and who are still on active duty. Further related to external validity, we consider that the tests can be used by physiotherapists with experience from musculoskeletal assessment and movement analysis since previous studies have found higher agreement for experienced physiotherapists compared to novels [23]. Practicing of tests with well-defined grading criteria are always important, especially for inexperienced therapists.

The attained kappa coefficients might have been enhanced by using grading criteria with more pronounced movement deviations rated as failed tests, as described by Luomajoki et al. [22]. We believe, however, as stated by Sahrmann [31], that subtle deviations in movement performances from what can be considered as optimal likely contribute to the development of pain. It is therefore important to make a careful observation of the movement control during the tests. Also, palpation during the test would likely enhance the kappa coefficients, since this adds information of movement control. However, with the design we used in which two PTs assessed the movements simultaneously, we hypothesized that their ratings would be interfered by simultaneous palpation. We further hypothesized that palpation during movements on test 1 can affect how the participant would move on test 2. The only test where palpation was allowed was on the Shoulder lateral rotation test because during the training phase the PTs found that visible scapular adductor muscle contraction affected the PTs’ perception of the amount of scapular adduction during the test. Despite the fact that we allowed palpation, the agreement of that test on the left side was moderate and on the right side was substantial (73.0%, kappa = 0.47 and 83.8%, kappa = 0.68, respectively) in the inter-rater study, but there were overlapping confidence intervals for the kappa coefficient. In the test-retest study, the kappa coefficients from the left and right sides showed different values in some of the included tests. This might reflect motor variability over time. We cannot compare our results to previous studies because results from the left and right side separately have not been reported. The present study included military personnel on their respective workplaces. Almost half of the personnel reported MSCI at present in any of the investigated regions. This fact could be seen as a strength since it reduces the risk of a skewed distribution of positive and negative test results in the kappa statistics. However, it has earlier shown that pain might influence movement control test result [37] and could thus influence the test-retest reliability. Future studies should investigate whether agreement in intra- and interrater reliability differ in subjects with and without MSCI.

### Clinical implications

In military settings, reliable tests are needed which are easy and efficient to use in clinical practice. Based on the PABAK results (>0.6), the authors propose to use the following tests when screening for biomechanically less advantageous movement patterns that might mechanically stress the tissues; Shoulder flexion, and rotation, Neck flexion in sitting and supine, Neck extension and rotation in sitting, Pelvic tilt, Forward lean and Single and Double knee extension tests. These tests can be considered reliable for assessing and evaluating movement patterns using two or more observers. The Pelvic tilt test and Single knee extension test are reliable in follow up situations (e.g. interventions). Objective tests such as the tests we included need to be reliable and valid for the results to be applied across different settings and populations. In view of our findings among military personnel, future studies should explore the reliability of these tests in various settings and subgroups of individuals (pain due to different underlying pan mechanisms) to document the clinical usefulness of the tests.

From a clinical perspective, we suggest not only to observe the movement control visually, but also to palpate the observed body parts as usual procedure in the clinic [21, 26, 33]. This will likely add more information regarding the quality of the examined movements during screening procedures of military personnel. The value of adding analyses of movement patterns to self-reported measurements should be investigated. Earlier studies have shown that using questionnaires and physical performance tests for screening of strength, endurance and flexibility deficits [10, 13], a positive result leads to further examination and relevant treatment and rehabilitation. To study the long-term effects of screening, longitudinal studies are needed to investigate the importance of poor movement control on the incidences of painful MSCI episodes among military personnel.

## Conclusion

In a military population, visual observations of movement control tests for neck and shoulder in sitting and standing, together with tests for thoracic, lumbar, and hip regions in sitting, could reliably be evaluated at the same occasion by two experienced PTs in this study. However, grading criteria for tests in supine and quadruped positions need to be further elaborated in the evaluation of movement control tests and factors affecting the lower test-retest reliability needs to be studied further.

## Supporting information

S1 Table Movement control tests. (PDF)

S1 File Data. (XLSX)
