# Supplementary material for: Inter-rater and test-retest reliability of movement control tests for the neck, shoulder, thoracic, lumbar, and hip regions in military personnel
Source: PLoS One. 2018 Sep 25;13(9):e0204552. doi: 10.1371/journal.pone.0204552 (PMC6155551; doi:10.1371/journal.pone.0204552)
Supplement: S1 Table — (PDF) [file pone.0204552.s001.pdf]

Appendix S1

Description of movement control tests with grading criteria used in the study.

Prior to each test, a short video of the test was shown to the participants together with verbal instructions. The participants repeated the movement three times to ensure familiarization with the movement to be tested, thereafter they performed the movement and the PTs rated them as either "optimal" or "non-optimal" movement patterns (i.e. "pass" or "fail" of the test). The tests have been presented earlier in text books [1-3]. Since this study didn´t include palpation in the rating of the tests, the grading criteria had to be slightly adapted to serve to the design of this study. In the case of a non-optimal rating, the PTs noted the reason according to the pre-defined grading criteria (see below). No feedback regarding test outcome was given during or after the test. The order of the tests was maintained throughout the study, beginning with tests involving standing followed by sitting, supine, and quadruped test positions (However, the tests are here presented according to body regions).

Shoulder flexion test
[1]

The purpose was to assess the ability to move the arm into flexion to about 180° with ≈60 °upward rotation and no excessive winging, excessive elevation/abduction/forward tilt/downward rotation of the scapula or medial rotation of the humerus.

Start position

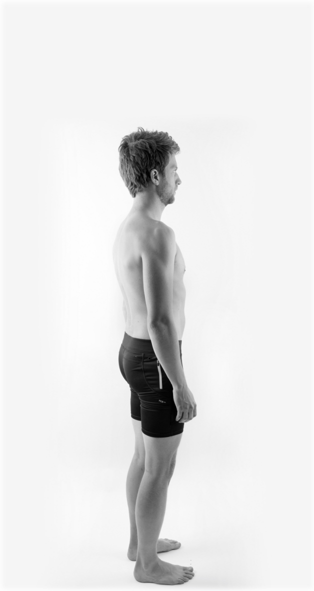

End position

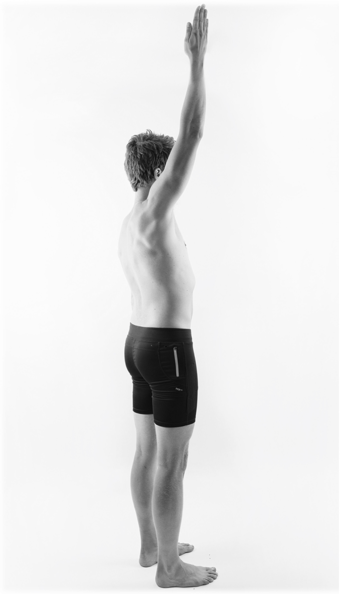

Participants were standing tall, the arm resting by the side with the scapula in a neutral position, and the glenohumeral joint in neutral rotation (palm in). From this position, participants were instructed to lift the right arm to full shoulder flexion, and then to return to the start position.

Grading criteria: The test was evaluated as passed if there was a flexion to about 180° with a sufficient upward rotation of the scapula (about 60°) such that the angulus inferior reached the mid-line of the lateral side of the thorax. It was evaluated as failed if there was winging, excessive elevation or abduction, forward tilt, or downward rotation of the scapula or if there was medial rotation of the humerus during shoulder flexion.

Shoulder extension test
Adapted from [3]

The purpose was to assess the ability to extend the arm to about 15° while retaining a neutral position of the scapula.

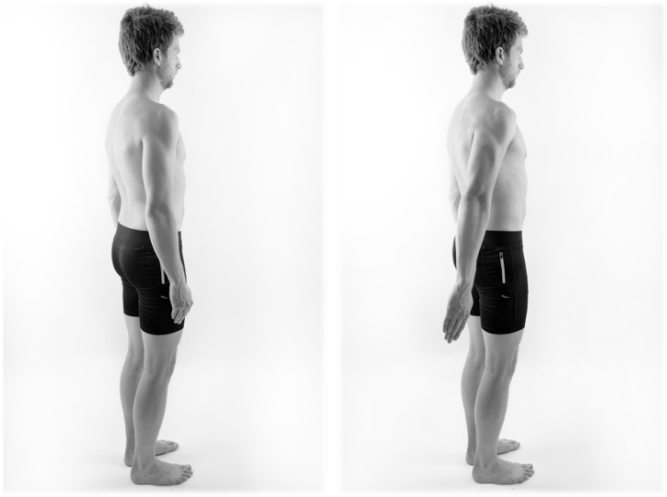

Participants were standing tall, with the arm resting by the side with the scapula in a neutral position and the glenohumeral joint in a neutral rotation (palm in). From this position, participants were instructed to extend the glenohumeral joint to about (-)15° while keeping the scapula neutral, and then to return to the start position.

Grading criteria: The test was evaluated as passed if there was an extension of the glenohumeral joint with only minimal movements of the scapula. It was evaluated as failed if there was winging, elevation, forward tilt, or downward rotation/adduction of the scapula or if there was medial rotation of the humerus during shoulder extension.

Shoulder lateral rotation test
Adapted from [3]

The purpose was to assess the ability to laterally rotate the shoulder to about 45° while retaining a neutral position of the scapula.

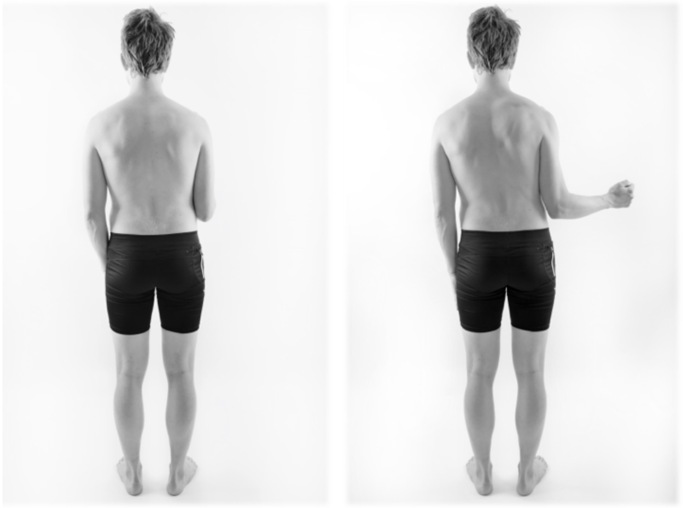

Participants were standing tall with the elbow by the side flexed 90°, the scapula in a neutral position, and the glenohumeral joint in a neutral rotation (palm in). From this position, participants were instructed to laterally rotate the glenohumeral joint to about 45° while keeping the scapula neutral, and then to return the arm back to the start position. The PT palpated the medial border of scapula during the test to assess if the neutral position was retained.

Grading criteria: The test was evaluated as passed if there was a lateral rotation of the glenohumeral joint to about 45° without movements of the scapula. It was evaluated as failed if there was forward tilt, downward rotation, or adduction of the scapula or if there was an anterior glide of the humeral head during shoulder lateral rotation.

Neck flexion in sitting test
Adapted from [2]

The purpose was to assess the ability to flex the neck to 45°–50° with contribution of both lower (≈35°) and upper cervical spine without cervical anterior translation/ diminished anterior sagittal plane rotation.

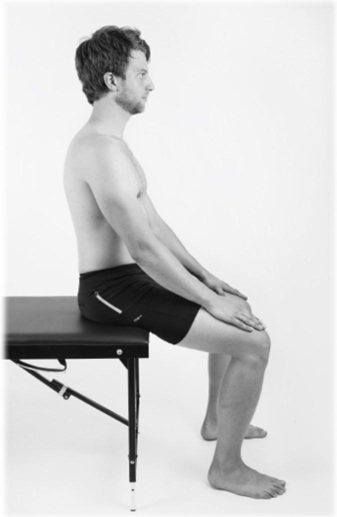

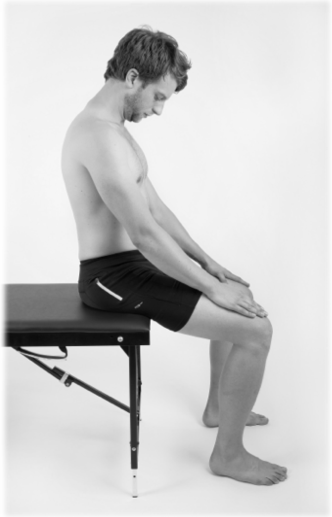

Participants were sitting on a bench with the feet on the floor, hands on the thighs and the neck, and back and shoulders in a neutral position. From this position, participants were instructed to flex their neck as far as possible, and then to return to the start position. The participants were instructed not to move the upper body during the movement.

Grading criteria: The test was evaluated as passed if the lower and upper cervical spine was contributing to flexion concurrently to 45°–50° (visually, whereof about 35° was performed in the lower cervical spine). It was evaluated as failed if there was increased or decreased flexion in the lower cervical spine, if there was increased or decreased flexion in the upper cervical spine, or if there was an anterior translation (head forward) of the head and cervical spine with diminished anterior sagittal plane rotation during neck flexion.

Neck extension in sitting test
Adapted from [2]

The purpose was to assess the ability to extend the neck to ≈85° with contribution of both lower (≈70°) and upper cervical spine without mid-cervical anterior translation.

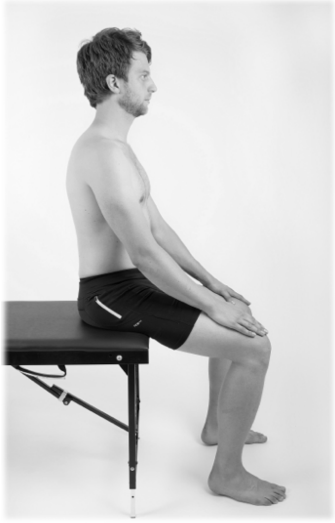

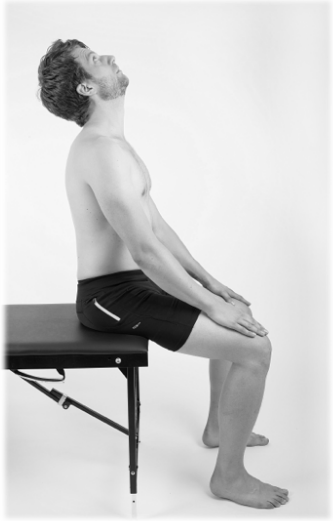

Participants were sitting on a bench with the feet on the floor, hands on the thighs and the neck, and the back and shoulders in a neutral position. From this position, participants were instructed to extend the neck as far as possible to look up at the ceiling, and then to return to the start position. The participants were instructed not to move the upper body or the shoulders during the movement and to keep the mouth closed.

Grading criteria: The test was evaluated as passed if the lower and upper cervical spine was contributing to extension concurrently to about 85° (visually, whereof about 70° was performed in the lower cervical spine). It was evaluated as failed if there was increased or decreased extension in the lower cervical spine, if there was increased or decreased extension in the upper cervical spine, or if there was a mid-cervical anterior translation (hinge) during neck extension.

Neck rotation test
Adapted from [3]

The purpose was to assess the ability to rotate the neck to about 70°–80° without concurrent neck or shoulder movements.

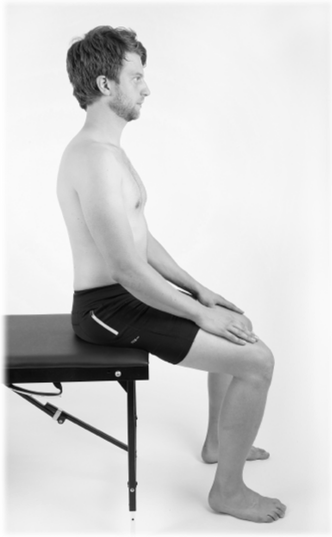

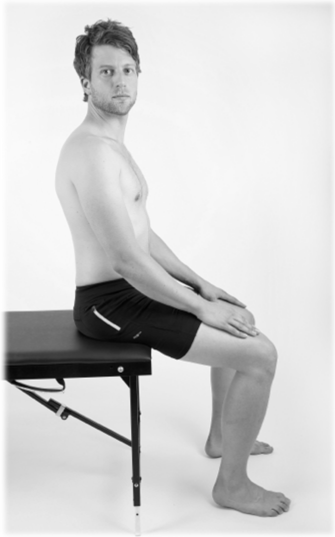

Participants were sitting on a bench with the feet on the floor, hands on the thighs and the neck, and the back and shoulders in a neutral position. From this position, participants were instructed to turn the head to the side as far as possible without bending the neck, and then to return to the start position. The participants were instructed not to move the upper body or the shoulders during the movement.

Grading criteria: The test was evaluated as passed if there was a cervical rotation to 70°–80° with no concurrent flexion (eyes kept horizontal) or extension or any shoulder movements. It was evaluated as failed if there were flexion, or lateral flexion of the neck or an excessive or early rotation in the thoracic spine or movements of the scapula during neck rotation.

Neck flexion in supine test
Adapted from [2]

The purpose was to assess the ability to smoothly flex the neck using all cervical segments without excessive anterior translation.

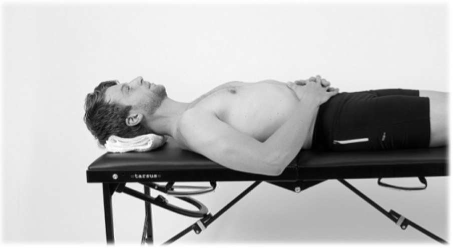

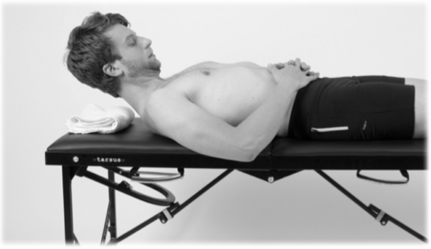

Participants were in a supine position on a bench, their arms lying on their belly with their head resting on a small towel to allow the neck to be in a neutral position. From this position, participants were instructed to flex their upper cervical spine by lightly holding the chin in followed by lifting the head off the bench without lifting the back, and then to return to the start position with a maintained upper cervical flexion.

Grading criteria: The test was evaluated as passed if the participant was able to smoothly flex their neck using all cervical segments, and then return to start position. The test was evaluated as failed if there was an excessive anterior translation in relation to the amount of anterior sagittal rotation or if the movement was jerky, indicating impaired muscle-recruitment pattern between intrinsic and extrinsic neck flexors.

Neck extension in quadruped test
Adapted from [2]

The purpose was to assess the ability to smoothly extend the cervical spine using all cervical segments without excessive posterior translation.

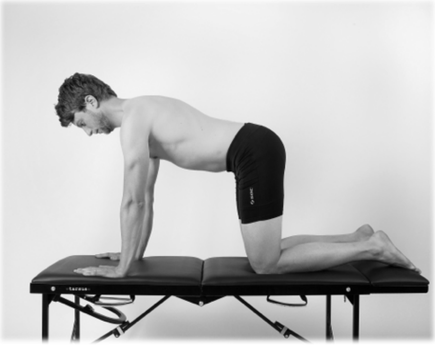

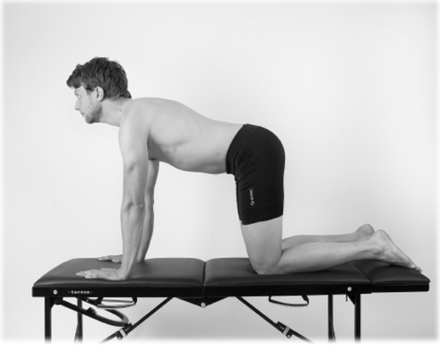

Participants were in a quadruped position, with their hands under the shoulders, the neck, back, and shoulders in a neutral position, and looking down between their hands. From this position, participants were instructed to extend their neck thus looking straight ahead, and then to return to the start position.

Grading criteria: The test was evaluated as passed if the participant was able to smoothly extend their cervical spine using all cervical segments and return to start position. It was evaluated as failed if there was an excessive posterior translation in relation to the amount of posterior sagittal rotation seen as overactive levator scapulae, indicating impaired muscle-recruitment pattern between intrinsic and extrinsic flexors.

Chest lift test
Adapted from [3]

The purpose was to assess the ability to extend the thoracic spine (lifting the chest) without anterior pelvic tilt and lumbar extension.

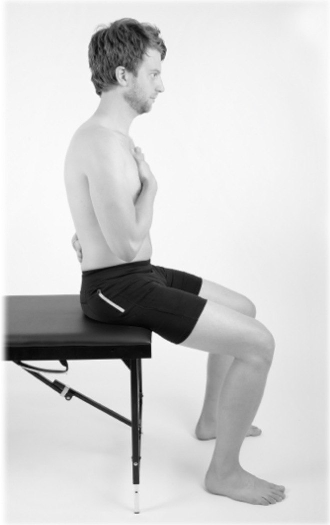

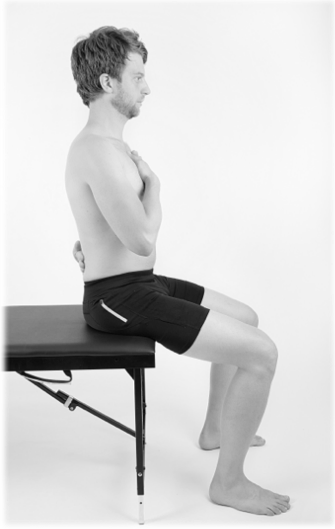

Participants were sitting on a bench with the feet on the floor, hands on the thighs and the neck, and low back and shoulders in a neutral position. The thoracic region was in slight flexion. From this position, participants were instructed to extend the thoracic region thus moving the sternum up and forward while keeping the lumbar region neutral, and then to return to the start position.

Grading criteria: The test was evaluated as passed if there was a thoracic extension lumbo-pelvic movements. It was evaluated as failed if there was anterior pelvic tilt and lumbar extension during thoracic extension.

Pelvic tilt test
Adapted from [3]

The purpose was to assess the ability to tilt the pelvis posteriorly without thoracic flexion.

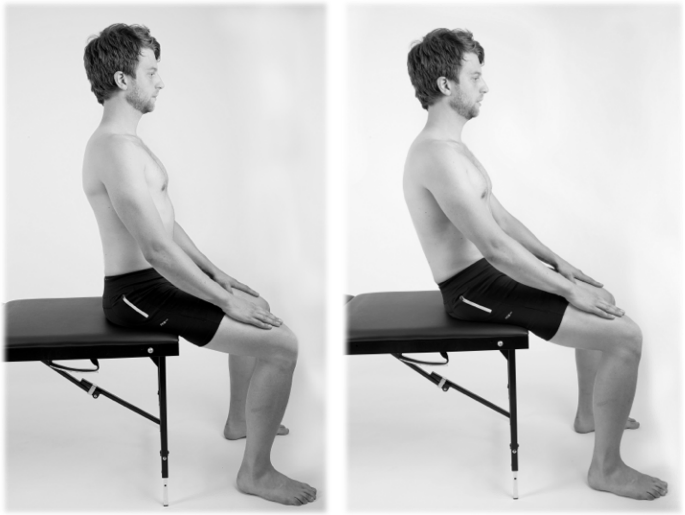

Participants were sitting on a bench with the feet on the floor, hands on the thighs and the neck, and the back and shoulders in a neutral position. From this position, participants were instructed to actively roll the pelvis backwards into posterior pelvic tilt while keeping the thoracic region neutral, and then to return to the start position.

Grading criteria: The test was evaluated as passed if there was a posterior pelvic tilt without thoracic flexion. It was evaluated as failed if there was a thoracic flexion during pelvic tilt.

Forward lean test
Adapted from [3]

The purpose was to assess the ability to flex the hip/lean forward to about 30° without lumbar flexion.

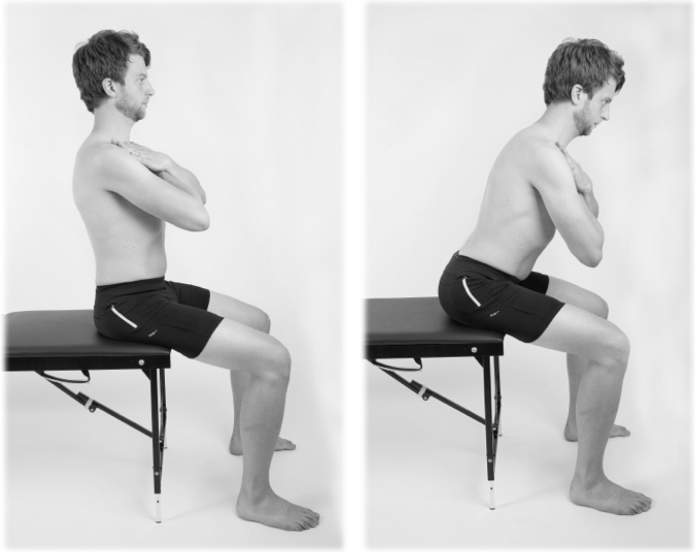

Participants were sitting on a bench with the feet on the floor, arms crossed on the chest and the neck, and the back and shoulders in a neutral position. From this position participants were instructed to flex their hips thus leaning their upper body forward to about 30° of flexion while keeping the lumbar region neutral, and then to return to the start position.

Grading criteria: The test was evaluated as passed if there was a hip flexion without lumbar flexion. It was evaluated as failed if there was a lumbar flexion or active extension during hip flexion.

Single knee extension test
Adapted from [3]

The purpose was to assess the ability to extend the knee to about 10°–15° from full extension without lumbar flexion or rotation.

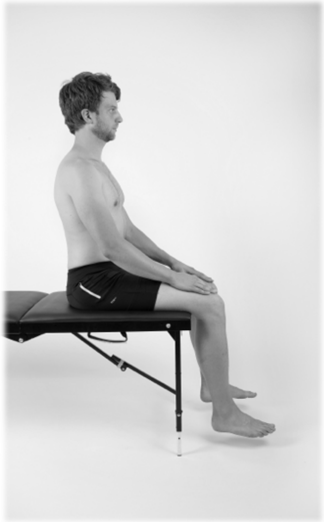

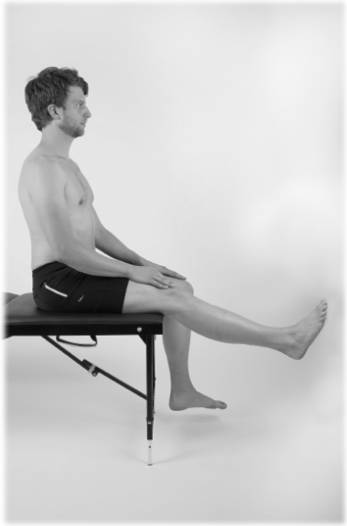

Participants were sitting on a bench with the feet unsupported, hands on the thighs and the neck, and the back and shoulders in a neutral position. From this position, participants were instructed to extend the knee to about 10°–15° from full extension while keeping the lumbar region neutral, and then to return to the start position.

Grading criteria: The test was evaluated as passed if the participant was able to extend the knee without lumbar flexion or rotation. It was evaluated as failed if there was lumbar flexion or rotation during knee extension.

Double knee extension
Adapted from [3]

The purpose was to assess the ability to extend both knees to about 10°–15° from full extension without lumbar flexion.

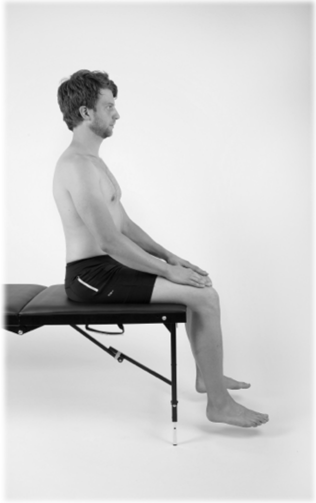

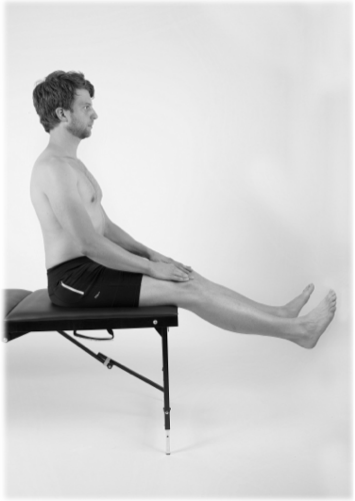

Participants were sitting on a bench with the feet unsupported, hands on the thighs and the neck, and the back and shoulders in neutral position. From this position, participants were instructed to extend both knees to about 10°–15° from full extension while keeping the lumbar region neutral, and then to return to the start position.

Grading criteria: The test was evaluated as passed if the participant was able to extend the knees without lumbar flexion. It was evaluated as failed if there was lumbar flexion during knee extension.

Leg lift test

Adapted from [1]

The purpose was to assess the ability to flex the hip joint to about 120° without lumbar flexion or posterior pelvic tilt.

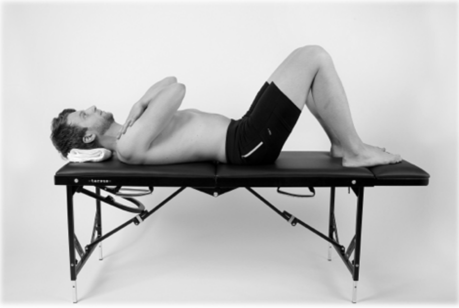

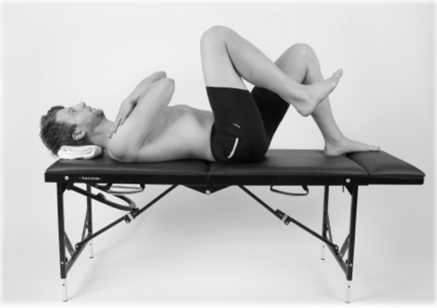

Participants were in a supine position on a bench, hips and knees flexed about 45° with their arms crossed on their chest and the head resting on a small towel. The PT first examined the passive range of motion in the hip. From this position, participants were instructed to flex the hip to about 120° while keeping the lumbo-pelvic region neutral, and then to return to the start position.

Grading criteria: The test was evaluated as passed if the hip was flexed to 120° without lumbar flexion and posterior pelvic tilt. It was evaluated as failed if there was lumbar flexion and/or posterior pelvic tilt during hip flexion.

Rocking forward test

Adapted from [3]

The purpose was to assess the ability to extend the hips to about 0° in quadruped position without lumbar extension.

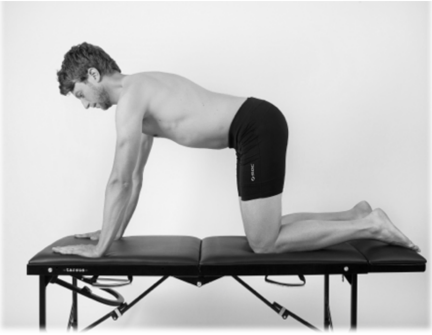

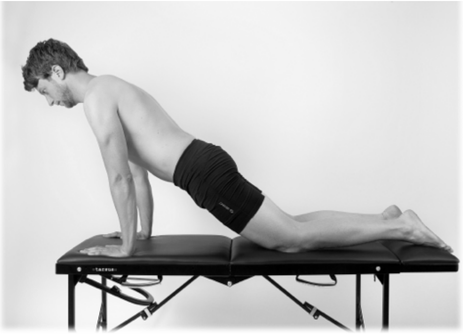

Participants were in a quadruped position, with their hands under their head, and the neck, back, and shoulders in a neutral position, looking down between their hands. From this position, participants were instructed to rock forward from the hips and shift their body weight forwards over their hands to about 0° of hip flexion while keeping the lumbar region neutral, and then to return to the start position.

Grading criteria: The test was evaluated as passed if the upper body and thighs were in line (about 0° of hip flexion) without lumbar extension. It was evaluated as failed if there was lumbar extension and/or anterior pelvic tilt during hip extension.

References

1. Sahrmann S. Diagnosis and treatment of movement impairment syndromes. St. Louis, Mo. ;: Mosby; 2002.
2. Sahrmann S. Movement system impairment syndromes of the extremities, cervical and thoracic spines. St. Louis, Mo: Mosby; 2011.
3. Comerford M. Kinetic control : the management of uncontrolled movement. In: Mottram S, editor. Rev. ed. ed. Chatswood, N.S.W.: Chatswood, N.S.W. : Elsevier Australia; 2012.
